# Supplementary material for: Side Effects of Kaolin and Bunch-Zone Leaf Removal on Generalist Predators in Vineyards
Source: Insects. 2023 Jan 25;14(2):126. doi: 10.3390/insects14020126 (PMC9966790; doi:10.3390/insects14020126)
Supplement: Supplementary file 1 [file insects-14-00126-s001.zip › insects-2120088-supplementary.pdf]

Table S1. Spiders collected with the drop cloth method in vineyards and years. For each taxon, label is reported as used in Figure 1.

Taxon and hunting strategy			Label	Vineyard A 2015	Vineyard A 2016	Vineyard B 2015	Vineyard C 2016	N.
Web-builders	Araneidae	*Araneus* sp. A juvenile	Ara.A	3	-	3	-	6
		Araneus sp. B	Ara.B	-	3	2	3	8
		Mangora acalypha (Walckenaer)	M.a.	1	3	13	3	20
		Nuctenea sp.	Nuc.	8	5	6	11	30
		Zilla diodia (Walckenaer)	Z.d.	-	-	1	-	1
		Araneidae sp. C	Ara.C	-	-	1	-	1
		Araneidae sp. D	Ara.D	-	-	1	-	1
		Araneidae juvenile	Ara.j.	-	-	5	-	5
		**Total Araneidae**		**12**	**11**	**32**	**17**	**72**
	Linyphiidae	Erigoninae, sp. A	Eri.A	-	3	-	8	11
		Erigoninae, sp. B	Eri.B	-	1	2	-	3
		Neriene sp.	Ner.	1	2	18	-	21
		Linyphiinae sp	Lin.	1	-	-	-	1
		**Total Linyphiidae**		**2**	**6**	**20**	**8**	**36**
	Theridiidae	*Theridion* sp.	The.	3	1	9	-	13
**Total web-builders**				**17**	**18**	**61**	**25**	**121**
Hunters	Thomisidae (ambushers)	*Runcinia grammica* (C.L. Koch)	R.g.	-	13	5	1	19
		Runcinia sp.	Run.	1	-	3		4
		Synema globosum (Fabricius)	S.g.	3	1	-	1	5
		Thomisus sp.	Tho.	7	1	10	-	18
		Xysticus sp.	Xys.	20	12	35	3	70
		**Total Thomisidae**		**31**	**27**	**53**	**5**	**116**
	Gnaphosidae (active)	*Drassodes* sp.	Dra.	-	3	12	1	16
		Gnaphosidae sp.	Gna.	4	-	-	-	4
		**Total Gnaphosidae**		**4**	**3**	**12**	**1**	**20**
	Miturgidae (active)	*Cheiracanthium* sp.	Che.	-	1	-	1	2
	Oxyopidae (active)	*Oxyopes lineatus* Latreille	O.l.	1	-	6	-	7
		Oxyopes sp.	Oxy.	12	2	13	13	40
		**Total Oxyopidae**		**13**	**2**	**19**	**13**	**47**
		Pseudeuophrys sp.	Pse.	-	-	1	-	1
	Salticidae (active)	*Pseudicius encarpatus* (Walckenaer)	P.e.	-	8	-	6	14
		Salticus scenicus (Clerck)	S.s.	-	-	1	6	7
		Salticidae juvenile	Sal.j.	5	4	9	3	21
		**Total Salticidae**		**5**	**12**	**11**	**15**	**43**
	Sparassidae (active)	*Micrommata virescens* (Clerck)	M.v.	-	2	4	-	6
**Total hunters**				**53**	**47**	**99**	**35**	**234**
**Total spiders**				**70**	**65**	**160**	**60**	**355**

Table S2. Predatory insects and spiders captured by yellow sticky traps in 2015 in Vineyards A and B.

Taxon	Vineyard A 2015	Vineyard B 2015	Total
Insects			
Aeolothrips sp. (Thysanoptera: Aeolothripidae)	1045	1185	2230
Scymninae (Coleoptera: Cocinellidae)	76	307	383
Coccinellidae non- Scymninae	53	121	174
Chrysoperla carnea s.l. (Neuroptera: Chrysopidae)	26	37	63
Orius sp. (Hemiptera: Anthocoridae)	29	31	60
**Total insects**	**1229**	**1681**	**2910**
Spiders			
Web-builders	93	127	220
Hunters	127	192	319
Gnaphosidae	5	12	17
Oxyopidae	11	36	47
Salticidae	43	44	87
Thomisidae	68	100	168
**Total spiders**	**220**	**319**	**539**
**Total predatory arthropods**	**1449**	**2000**	**3449**
